# 1,3-Bis(1-methyl-1*H*-benzimidazol-2-yl)-2-oxapropane

**DOI:** 10.1107/S1600536811017922

**Published:** 2011-05-20

**Authors:** Fan Kou, Bin Liu, Ying Bai, Jin Kong, Huilu Wu

**Affiliations:** a School of Chemical and Biological Engineering, Lanzhou Jiaotong University, Lanzhou 730070, People’s Republic of China

## Abstract

In the title mol­ecule, C_18_H_18_N_4_O, the dihedral angle between the mean planes of the two benzimidazole ring systems is 61.5 (1)°.

## Related literature

For biological applications of benzimidazoles and bis-benzimidazoles, see: Horton *et al.* (2003 ▶); Holland & Tolman (2000 ▶). For related structures, see: Chen *et al.* (2009 ▶); Wu *et al.* (2009 ▶).
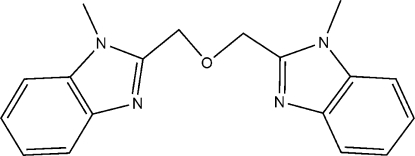


## Experimental

### 

#### Crystal data

C_18_H_18_N_4_O
*M* *_r_* = 306.36
Monoclinic, 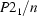

*a* = 6.634 (6) Å
*b* = 16.217 (15) Å
*c* = 14.457 (13) Å
β = 101.102 (10)°
*V* = 1526 (2) Å^3^
*Z* = 4
Mo *K*α radiation
μ = 0.09 mm^−1^
*T* = 296 K
0.26 × 0.24 × 0.21 mm

#### Data collection

Bruker APEXII CCD diffractometer
Absorption correction: multi-scan (*SADABS*; Sheldrick, 1996 ▶) *T*_min_ = 0.978, *T*_max_ = 0.982
10239 measured reflections
2668 independent reflections
1846 reflections with *I* > 2σ(*I*)
*R*_int_ = 0.057

#### Refinement

*R*[*F* ^2^ > 2σ(*F* ^2^)] = 0.063
*wR*(*F* ^2^) = 0.169
*S* = 1.05
2668 reflections
210 parameters
H-atom parameters constrained
Δρ_max_ = 0.19 e Å^−3^
Δρ_min_ = −0.19 e Å^−3^

### 

Data collection: *APEX2* (Bruker, 2007 ▶); cell refinement: *SAINT* (Bruker, 2007 ▶); data reduction: *SAINT*; program(s) used to solve structure: *SHELXS97* (Sheldrick, 2008 ▶); program(s) used to refine structure: *SHELXL97* (Sheldrick, 2008 ▶); molecular graphics: *SHELXTL* (Sheldrick, 2008 ▶); software used to prepare material for publication: *SHELXTL*.

## Supplementary Material

Crystal structure: contains datablocks global, I. DOI: 10.1107/S1600536811017922/lh5249sup1.cif

Structure factors: contains datablocks I. DOI: 10.1107/S1600536811017922/lh5249Isup2.hkl

Supplementary material file. DOI: 10.1107/S1600536811017922/lh5249Isup3.cml

Additional supplementary materials:  crystallographic information; 3D view; checkCIF report

## Figures and Tables

**Fig. 1.**
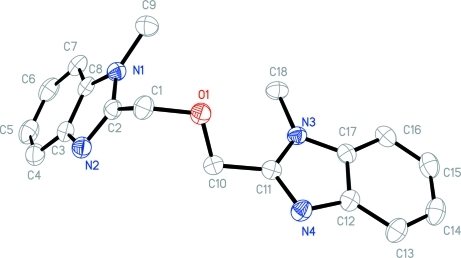
The molecular structure of the title compound. Displacement ellipsoids are drawn at the 30% probability level. H atoms have been omitted for clarity.

## supplementary crystallographic information

### Comment

The benzimidazole core is of interest because of its diverse biological activies, and it is a well known in medicinal chemistry (Horton *et al.*, 2003). In bioinorganic chemistry, bis-benzimidazoles have been used extensively to help model the active sites of metalloproteins (Holland & Tolman, 2000). The crystal structures of 1,3-bis(1-benzimidazol-2-yl)-2-oxopropane and 1,3-bis(1-benzylbenzimidazol-2-yl)-2-oxopropane have been reported previously (Wu *et al.* 2009; Chen *et al.*. 2009). The molecular structure of the title compound is shown in Fig.1. The dihedral angle between the mean planes of the two benzimidazole ring systems is 61.5 (1) Å.

### Experimental

A solution of 5.56 g (20 mmol) of 1.3-bis(benzimidazol-2-yl)-2-oxpropane with 1.56 g (40 mmol) potassium in the 150 ml tetrahydrofuran followed by addition 5.68 g (40 mmol) methyl iodide was concentrated and recrystallized from methanol, formimg white blocks suitable for X-ray diffraction studies.(found: C, 70.50; H, 5.83; N, 18.34. Calad.: C, 70.57; H, 5.92; N, 18.29)

### Refinement

All H atoms were found in difference electron maps and were subsequently refined in a riding-model approximation with C—H distances ranging from 0.93 to 0.97 Å and *U*_iso_(H) = 1.2 *U*_eq_(C) or *U*_iso_(H) = 1.5*U*_eq_(C_methyl_).

### Crystal data

**Table:**

C_18_H_18_N_4_O	*F*(000) = 648
*M**_r_* = 306.36	*D*_x_ = 1.333 Mg m^−^^3^
Monoclinic, *P*2_1_/*n*	Mo *K*α radiation, λ = 0.71073 Å
Hall symbol: -P 2yn	Cell parameters from 1941 reflections
*a* = 6.634 (6) Å	θ = 2.5–23.0°
*b* = 16.217 (15) Å	µ = 0.09 mm^−^^1^
*c* = 14.457 (13) Å	*T* = 296 K
β = 101.102 (10)°	Block, white
*V* = 1526 (2) Å^3^	0.26 × 0.24 × 0.21 mm
*Z* = 4	

### Data collection

**Table:**

Bruker APEXII CCD diffractometer	2668 independent reflections
Radiation source: fine-focus sealed tube	1846 reflections with *I* > 2σ(*I*)
graphite	*R*_int_ = 0.057
ω scans	θ_max_ = 25.0°, θ_min_ = 2.5°
Absorption correction: multi-scan (*SADABS*; Sheldrick, 1996)	*h* = −7→7
*T*_min_ = 0.978, *T*_max_ = 0.982	*k* = −17→19
10239 measured reflections	*l* = −17→17

### Refinement

**Table:**

Refinement on *F*^2^	Secondary atom site location: difference Fourier map
Least-squares matrix: full	Hydrogen site location: inferred from neighbouring sites
*R*[*F*^2^ > 2σ(*F*^2^)] = 0.063	H-atom parameters constrained
*wR*(*F*^2^) = 0.169	*w* = 1/[σ^2^(*F*_o_^2^) + (0.062*P*)^2^ + 1.2125*P*] where *P* = (*F*_o_^2^ + 2*F*_c_^2^)/3
*S* = 1.05	(Δ/σ)_max_ < 0.001
2668 reflections	Δρ_max_ = 0.19 e Å^−^^3^
210 parameters	Δρ_min_ = −0.19 e Å^−^^3^
0 restraints	Extinction correction: *SHELXL97* (Sheldrick, 2008), Fc^*^=kFc[1+0.001xFc^2^λ^3^/sin(2θ)]^-1/4^
Primary atom site location: structure-invariant direct methods	Extinction coefficient: 0.0025 (4)

### Special details

**Table:**

Geometry. All e.s.d.'s (except the e.s.d. in the dihedral angle between two l.s. planes) are estimated using the full covariance matrix. The cell e.s.d.'s are taken into account individually in the estimation of e.s.d.'s in distances, angles and torsion angles; correlations between e.s.d.'s in cell parameters are only used when they are defined by crystal symmetry. An approximate (isotropic) treatment of cell e.s.d.'s is used for estimating e.s.d.'s involving l.s. planes.
Refinement. Refinement of *F*^2^ against ALL reflections. The weighted *R*-factor *wR* and goodness of fit *S* are based on *F*^2^, conventional *R*-factors *R* are based on *F*, with *F* set to zero for negative *F*^2^. The threshold expression of *F*^2^ > σ(*F*^2^) is used only for calculating *R*-factors(gt) *etc*. and is not relevant to the choice of reflections for refinement. *R*-factors based on *F*^2^ are statistically about twice as large as those based on *F*, and *R*- factors based on ALL data will be even larger.

### Fractional atomic coordinates and isotropic or equivalent isotropic displacement parameters (Å^2^)

**Table:**

	*x*	*y*	*z*	*U*_iso_*/*U*_eq_	
O1	0.1874 (3)	0.23663 (13)	0.39791 (14)	0.0471 (6)	
N3	0.4241 (4)	0.11107 (15)	0.52270 (17)	0.0410 (6)	
N1	0.2920 (4)	0.23299 (15)	0.19655 (17)	0.0439 (6)	
C11	0.2170 (4)	0.12133 (18)	0.5023 (2)	0.0407 (7)	
N2	−0.0371 (4)	0.19270 (17)	0.16948 (18)	0.0489 (7)	
C12	0.2789 (5)	0.05940 (18)	0.6348 (2)	0.0427 (7)	
C8	0.2615 (5)	0.18872 (19)	0.1131 (2)	0.0437 (8)	
N4	0.1227 (4)	0.09232 (16)	0.56732 (18)	0.0467 (7)	
C17	0.4691 (5)	0.07179 (18)	0.6089 (2)	0.0410 (7)	
C3	0.0574 (5)	0.1645 (2)	0.0974 (2)	0.0463 (8)	
C2	0.1090 (5)	0.23293 (19)	0.2256 (2)	0.0424 (7)	
C16	0.6514 (5)	0.0441 (2)	0.6624 (2)	0.0524 (9)	
H16	0.7768	0.0531	0.6444	0.063*	
C18	0.5751 (5)	0.1358 (2)	0.4666 (2)	0.0505 (8)	
H18A	0.5239	0.1228	0.4015	0.076*	
H18B	0.7014	0.1067	0.4882	0.076*	
H18C	0.5990	0.1940	0.4731	0.076*	
C13	0.2710 (6)	0.0182 (2)	0.7183 (2)	0.0553 (9)	
H13	0.1466	0.0102	0.7377	0.066*	
C10	0.1078 (5)	0.1576 (2)	0.4120 (2)	0.0482 (8)	
H10A	0.1235	0.1217	0.3602	0.058*	
H10B	−0.0376	0.1620	0.4129	0.058*	
C15	0.6381 (6)	0.0023 (2)	0.7440 (2)	0.0610 (10)	
H15	0.7574	−0.0179	0.7818	0.073*	
C1	0.0810 (5)	0.2749 (2)	0.3144 (2)	0.0494 (8)	
H1A	0.1279	0.3315	0.3132	0.059*	
H1B	−0.0643	0.2763	0.3162	0.059*	
C4	−0.0213 (6)	0.1176 (2)	0.0180 (2)	0.0612 (10)	
H4A	−0.1579	0.1007	0.0055	0.073*	
C7	0.3946 (6)	0.1688 (2)	0.0531 (2)	0.0550 (9)	
H7	0.5310	0.1858	0.0645	0.066*	
C5	0.1093 (7)	0.0971 (2)	−0.0410 (2)	0.0675 (11)	
H5	0.0601	0.0654	−0.0942	0.081*	
C9	0.4856 (5)	0.2688 (2)	0.2415 (3)	0.0614 (10)	
H9A	0.4693	0.2959	0.2986	0.092*	
H9B	0.5290	0.3082	0.1998	0.092*	
H9C	0.5871	0.2261	0.2559	0.092*	
C6	0.3114 (7)	0.1222 (2)	−0.0240 (2)	0.0657 (11)	
H6	0.3943	0.1070	−0.0662	0.079*	
C14	0.4501 (6)	−0.0104 (2)	0.7712 (2)	0.0616 (10)	
H14	0.4465	−0.0391	0.8266	0.074*	

### Atomic displacement parameters (Å^2^)

**Table:**

	*U*^11^	*U*^22^	*U*^33^	*U*^12^	*U*^13^	*U*^23^
O1	0.0496 (12)	0.0464 (13)	0.0446 (12)	−0.0009 (10)	0.0075 (10)	0.0015 (10)
N3	0.0392 (14)	0.0429 (14)	0.0424 (14)	−0.0022 (11)	0.0119 (11)	−0.0009 (11)
N1	0.0427 (15)	0.0459 (15)	0.0439 (15)	−0.0012 (12)	0.0102 (11)	0.0036 (12)
C11	0.0399 (17)	0.0407 (17)	0.0426 (17)	−0.0002 (14)	0.0106 (13)	−0.0045 (14)
N2	0.0466 (16)	0.0534 (17)	0.0468 (15)	0.0040 (13)	0.0090 (12)	0.0063 (13)
C12	0.0520 (19)	0.0372 (17)	0.0407 (17)	0.0057 (14)	0.0134 (14)	−0.0051 (13)
C8	0.053 (2)	0.0408 (18)	0.0398 (17)	0.0079 (15)	0.0153 (14)	0.0134 (14)
N4	0.0443 (15)	0.0505 (16)	0.0479 (15)	0.0059 (12)	0.0151 (12)	0.0048 (12)
C17	0.0447 (17)	0.0352 (17)	0.0423 (17)	0.0015 (14)	0.0063 (13)	−0.0076 (13)
C3	0.0506 (19)	0.0472 (19)	0.0399 (17)	0.0044 (15)	0.0057 (14)	0.0075 (14)
C2	0.0401 (17)	0.0438 (18)	0.0433 (17)	0.0076 (14)	0.0075 (14)	0.0079 (14)
C16	0.0474 (19)	0.048 (2)	0.058 (2)	0.0011 (15)	0.0009 (15)	−0.0067 (16)
C18	0.0440 (18)	0.053 (2)	0.059 (2)	−0.0054 (16)	0.0199 (15)	−0.0034 (16)
C13	0.066 (2)	0.054 (2)	0.050 (2)	0.0105 (17)	0.0214 (17)	0.0032 (16)
C10	0.0456 (18)	0.054 (2)	0.0453 (18)	−0.0070 (16)	0.0084 (14)	0.0050 (15)
C15	0.070 (2)	0.048 (2)	0.057 (2)	0.0137 (18)	−0.0074 (18)	−0.0001 (17)
C1	0.055 (2)	0.0468 (19)	0.0469 (18)	0.0086 (16)	0.0113 (15)	0.0040 (15)
C4	0.072 (2)	0.055 (2)	0.052 (2)	−0.0047 (19)	0.0003 (18)	0.0031 (17)
C7	0.065 (2)	0.052 (2)	0.052 (2)	0.0065 (17)	0.0228 (17)	0.0138 (17)
C5	0.102 (3)	0.059 (2)	0.042 (2)	0.002 (2)	0.014 (2)	−0.0020 (17)
C9	0.051 (2)	0.070 (2)	0.065 (2)	−0.0092 (18)	0.0124 (17)	0.0022 (19)
C6	0.095 (3)	0.060 (2)	0.050 (2)	0.014 (2)	0.032 (2)	0.0097 (18)
C14	0.089 (3)	0.049 (2)	0.0462 (19)	0.014 (2)	0.0103 (19)	0.0023 (16)

### Geometric parameters (Å, °)

**Table:**

O1—C10	1.416 (4)	C18—H18A	0.9600
O1—C1	1.419 (4)	C18—H18B	0.9600
N3—C11	1.359 (4)	C18—H18C	0.9600
N3—C17	1.380 (4)	C13—C14	1.364 (5)
N3—C18	1.461 (4)	C13—H13	0.9300
N1—C2	1.359 (4)	C10—H10A	0.9700
N1—C8	1.385 (4)	C10—H10B	0.9700
N1—C9	1.445 (4)	C15—C14	1.394 (5)
C11—N4	1.313 (4)	C15—H15	0.9300
C11—C10	1.486 (4)	C1—H1A	0.9700
N2—C2	1.311 (4)	C1—H1B	0.9700
N2—C3	1.392 (4)	C4—C5	1.369 (5)
C12—N4	1.386 (4)	C4—H4A	0.9300
C12—C13	1.390 (4)	C7—C6	1.372 (5)
C12—C17	1.398 (4)	C7—H7	0.9300
C8—C3	1.386 (4)	C5—C6	1.377 (5)
C8—C7	1.389 (4)	C5—H5	0.9300
C17—C16	1.380 (4)	C9—H9A	0.9600
C3—C4	1.393 (5)	C9—H9B	0.9600
C2—C1	1.497 (4)	C9—H9C	0.9600
C16—C15	1.378 (5)	C6—H6	0.9300
C16—H16	0.9300	C14—H14	0.9300
			
C10—O1—C1	112.4 (2)	C12—C13—H13	120.8
C11—N3—C17	106.7 (2)	O1—C10—C11	110.6 (2)
C11—N3—C18	128.2 (3)	O1—C10—H10A	109.5
C17—N3—C18	125.1 (3)	C11—C10—H10A	109.5
C2—N1—C8	106.2 (2)	O1—C10—H10B	109.5
C2—N1—C9	129.1 (3)	C11—C10—H10B	109.5
C8—N1—C9	124.6 (3)	H10A—C10—H10B	108.1
N4—C11—N3	113.7 (3)	C16—C15—C14	121.5 (3)
N4—C11—C10	123.5 (3)	C16—C15—H15	119.2
N3—C11—C10	122.7 (3)	C14—C15—H15	119.2
C2—N2—C3	103.9 (3)	O1—C1—C2	114.0 (3)
N4—C12—C13	130.4 (3)	O1—C1—H1A	108.7
N4—C12—C17	110.3 (3)	C2—C1—H1A	108.7
C13—C12—C17	119.3 (3)	O1—C1—H1B	108.7
N1—C8—C3	105.2 (3)	C2—C1—H1B	108.7
N1—C8—C7	131.3 (3)	H1A—C1—H1B	107.6
C3—C8—C7	123.4 (3)	C5—C4—C3	117.7 (4)
C11—N4—C12	104.3 (3)	C5—C4—H4A	121.2
N3—C17—C16	132.2 (3)	C3—C4—H4A	121.2
N3—C17—C12	105.0 (3)	C6—C7—C8	115.5 (3)
C16—C17—C12	122.8 (3)	C6—C7—H7	122.3
C8—C3—N2	110.6 (3)	C8—C7—H7	122.3
C8—C3—C4	119.2 (3)	C4—C5—C6	121.9 (4)
N2—C3—C4	130.2 (3)	C4—C5—H5	119.0
N2—C2—N1	114.1 (3)	C6—C5—H5	119.0
N2—C2—C1	123.9 (3)	N1—C9—H9A	109.5
N1—C2—C1	122.0 (3)	N1—C9—H9B	109.5
C15—C16—C17	116.6 (3)	H9A—C9—H9B	109.5
C15—C16—H16	121.7	N1—C9—H9C	109.5
C17—C16—H16	121.7	H9A—C9—H9C	109.5
N3—C18—H18A	109.5	H9B—C9—H9C	109.5
N3—C18—H18B	109.5	C7—C6—C5	122.3 (3)
H18A—C18—H18B	109.5	C7—C6—H6	118.9
N3—C18—H18C	109.5	C5—C6—H6	118.9
H18A—C18—H18C	109.5	C13—C14—C15	121.4 (3)
H18B—C18—H18C	109.5	C13—C14—H14	119.3
C14—C13—C12	118.4 (3)	C15—C14—H14	119.3
C14—C13—H13	120.8		

## References

[bb1] Bruker (2007). *APEX2* and *SAINT* Bruker AXS Inc., Madison, Wisconsin, USA.

[bb2] Chen, Y., Guo, J., Yun, R. & Wu, H. (2009). *Acta Cryst.* E**65**, o948. 10.1107/S1600536809011507 PMC2977649 21583992

[bb3] Holland, P. L. & Tolman, W. B. (2000). *J. Am. Chem. Soc* **122**, 6331–6332

[bb4] Horton, D. A., Bourne, G. T. & Smythe, M. L. (2003). *Chem. Rev* **103**, 893–930. 10.1021/cr020033s 12630855

[bb5] Sheldrick, G. M. (1996). *SADABS* University of Göttingen, Germany.

[bb6] Sheldrick, G. M. (2008). *Acta Cryst.* A**64**, 112–122. 10.1107/S0108767307043930 18156677

[bb7] Wu, H., Yun, R., Wang, K., Huang, X. & Sun, Q. (2009). *Acta Cryst.* E**65**, o1014. 10.1107/S1600536809012781 PMC2977701 21583837

